# Supplementary material for: Emerging Role of Decoy Receptor-2 as a Cancer Risk Predictor in Oral Potentially Malignant Disorders
Source: Int J Mol Sci. 2023 Sep 21;24(18):14382. doi: 10.3390/ijms241814382 (PMC10531848; doi:10.3390/ijms241814382)
Supplement: Supplementary file 1 [file ijms-24-14382-s001.zip › ijms-2612360-supplementary.pdf]

SUPPLEMENTARY INFORMATION

Supplementary Table S1. Evolution of the premalignant lesions in relation to histopathological diagnosis using WHO binary dysplasia grading.

**Characteristics**	**No. cases (%)**	**Progression to OSCC [No. cases (%)]**	***p***
Histopathological diagnosis			
Without dysplasia	47 (78)	5 (11)	< 0.0001
Low-grade dysplasia	5 (8)	3 (60)	
High-grade dysplasia	3 (5)	3 (100)	

Chi-square test

Supplementary Table S2. Relationship between binary histological dysplasia grading and expression of Ki67, p16, DcR2 and DEC1.

**Protein expression**	**Histological grade of epithelial dysplasia**			***p***
	**Absent**	**Low-grade dysplasia**	**High-grade dysplasia**	
Ki67 expression (% of positive epithelial cells)				
Mild (score 0)	30 (63.8)	2 (28.6)	0 (0.0)	0.001
Moderate (score 1)	17 (36.2)	5 (71.4)	5 (83.3)	
Strong (score 2)	0 (0.0)	0 (0.0)	1 (16.7)	
Ki67 expression (epithelial distribution)				
Restricted to basal third (score 0)	35 (74.5)	3 (42.9)	0 (0.0)	<0.0001
Above basal third (score 1)	12 (25.5)	4 (57.1)	6 (100.0)	
p16 epithelial expression				
Negative (score 0)	41 (87.2)	7 (100)	4 (66.7)	0.218
Positive (score 1)	6 (12.8)	0 (0)	2 (33.3)	
DcR2 expression (% of positive epithelial cells)				
Mild (score 0)	44 (93.6)	4 (57.1)	3 (50.0)	0.002
Moderate (score 1)	2 (4.3)	3 (42.9)	5 (50.0)	
Strong (score 2)	1 (2.1)	0 (0.0)	0 (0.0)	
Nuclear DEC1 expression (epithelial distribution)				
No expression (score 0)	6 (13.6)	1 (14.2)	0 (0.0)	0.076
Restricted to basal layer (score 1)	27 (61.4)	3 (42.9)	1 (16.7)	
Suprabasal layer (score 2)	11 (25.0)	3 (42.9)	5 (83.3)	
Cytoplasmic DEC1 expression (epithelial distribution)				
No expression (score 0)	26 (59.1)	2 (28.6)	2 (33.3)	0.005
Restricted to basal layer (score 1)	16 (36.4)	2 (28.6)	1 (16.7)	
Suprabasal layer (score 2)	2 (4.5)	3 (42.9)	3 (50.0)	

Supplementary Table S3. Antibodies used in the immunohistochemical analysis of protein expression.

Protein	Primary antibody (manufacturer)	Antibody Dilution
Ki67	Mouse Monoclonal Clone MIB-1 (Dako)	Prediluted
p16	Mouse Monoclonal (CINtec® Histology)	Prediluted
DcR2	Rabbit Monoclonal (Abcam)	1:1000
DEC1	Rabbit, affinity isolated (Sigma)	1:200
